# Growth hormone (GH) replacement decreases serum total and LDL-cholesterol in hypopituitary patients on maintenance HMG CoA reductase inhibitor (statin) therapy

**DOI:** 10.1111/j.1365-2265.2007.02935.x

**Published:** 2007-10

**Authors:** John P Monson, Peter Jönsson, Maria Koltowska-Häggström, Ione Kourides

**Affiliations:** * Centre for Clinical Endocrinology, St Bartholomew's Hospital, William Harvey Research Institute, Queen Mary University of London UK; † Medical Outcomes, Pfizer Endocrine Care Sollentuna, Sweden; ‡ Department of Pharmacy, Uppsala University Uppsala, Sweden; § Pfizer Endocrine Care, Pfizer Inc New York, USA

## Abstract

**Objective:**

Adult onset GH deficiency (GHD) is characterized by abnormalities of serum lipoprotein profiles and GH replacement results in favourable alterations in serum total and low density lipoprotein (LDL)-cholesterol. Preliminary evidence has indicated that the effect of GH replacement in this respect may be additive to that of HMG CoA reductase inhibitor (statin) therapy. We have examined this possibility during prospective follow-up of adult onset hypopituitary patients enrolled in KIMS (Pfizer International Metabolic Database), a pharmacoepidemiological study of GH replacement in adult hypopituitary patients.

**Design:**

Lipoprotein profiles were measured centrally at baseline and after 12 months GH replacement therapy.

**Patients:**

Sixty-one hypopituitary patients (30 male, 31 female) on maintenance statin therapy (mean 2·5 ± 2·7 SD years before GH) (statin group – SG) and 1247 (608 male, 639 female) patients not on hypolipidaemic therapy (nonstatin group – NSG) were studied. All patients were naïve or had not received GH replacement during the 6 months prior to study. Patients who developed diabetes mellitus during the first year of GH therapy or in the subsequent year and those with childhood onset GHD were excluded from this analysis. An established diagnosis of diabetes mellitus was present in 18% SG and 4·4% NSG at baseline.

**Measurements:**

Serum concentrations of total, high density lipoprotein (HDL)-cholesterol, triglycerides and IGF-I were measured centrally in all patients and LDL-cholesterol was estimated using Friedewald's formula.

**Results:**

The relative frequency of various statin use was simvastatin 52% (15·8 ± 8·1 mg, mean ± SD), atorvastatin 30% (14·4 ± 7·8 mg), pravastatin 9·8% (31·6 mg ± 13·9 mg), lovastatin 6·6% (17·5 ± 5 mg) and fluvastatin 1·6% (40 mg). Baseline serum total and LDL-cholesterol (mean ± SD) were 5·2 ± 1·4 and 3·1 ± 1·3 mmol/l in SG and 5·8 ± 1·2 and 3·7 ± 1·0 mmol/l in NSG, respectively (*P* < 0·0001, SG *vs*. NSG). After 12 months GH replacement (SG: 0·32 ± 0·17 mg/day; NSG: 0·38 ± 0·1 mg/day) serum total and LDL-cholesterol decreased by a mean (±SD) of 0·48 (± 1·25) mmol/l (*P* < 0·0004) and 0·53 (± 1·08) mmol/l (*P* < 0·0001) in SG and by 0·30 (± 0·89) mmol/l (*P* < 0·0001) and 0·28 (± 0·80) mmol/l (*P* < 0·0001) in NSG, respectively. There were no significant changes in HDL-cholesterol or triglycerides in either group (SG *vs*. NSG: NS). A relationship between LDL-cholesterol at baseline and the decrease in LDL-cholesterol after 12 months GH was evident in both groups (SG: *R* = –0·54, *P* < 0·001; NSG: *R* = –0·4, *P* < 0·001) and a similar relationship for cholesterol was observed.

**Conclusions:**

These data indicate that GH replacement exerts additional beneficial effects on lipoprotein profiles in patients on maintenance statin therapy, confirming that the effects of these interventions are complementary rather than exclusive.

## Introduction

A number of retrospective studies have demonstrated that hypopituitarism is associated with a significant increase in standardized mortality ratio, particularly in females^1^–^4^ and these findings have been confirmed in a subsequent prospective multicentre study.^5^ The increase in mortality appears to be particularly related to cardiovascular and cerebrovascular disease.^1^,^3^–^5^ In all of these publications a unifying characteristic was the high prevalence of untreated growth hormone deficiency (GHD), with other deficiencies conventionally replaced. This raises the possibility that the GHD state may predispose to cardiovascular disease although other factors including aetiology (especially craniopharyngioma) and untreated oestrogen deficiency are likely to be additional and independent factors.^5^ Consistent with a causal role for GHD in the pathogenesis of cardiovascular disease, such deficiency is associated with an increased prevalence of cardiovascular risk factors including increased concentrations of total and low density lipoprotein (LDL)-cholesterol, increased central adiposity, insulin resistance and glucose intolerance. Furthermore, these abnormalities are significantly improved by growth hormone replacement therapy.^6^–^14^ Additional evidence favouring a specific contribution from GHD to adverse cardiovascular risk comes from studies comparing baseline characteristics and the effects of GH replacement in patients with isolated GHD in comparison with more generalized pituitary failure.^15^,^16^

The beneficial effects of GH replacement on serum lipoprotein profiles are particularly evident in relation to LDL-cholesterol, the durable decrement in which accounts for the changes in serum total cholesterol observed.^13^ Subtle changes in high density lipoprotein (HDL)-cholesterol may occur with prolonged treatment and improvements in serum triglycerides may be evident in specific aetiological groups and in patients with the highest pretreatment levels.^17^ The efficacy of 3-hydroxy-3-methylglutaryl coenzyme A (HMG CoA) reductase inhibitor (statin) therapy in lowering serum LDL-cholesterol concentrations begs the question as to whether there may be an additive effect of these agents and GH replacement in GHD hypopituitary patients. In terms of respective mechanism of action on cholesterol metabolism, such an effect would be predicted and has indeed been observed in a small number of patients in a single-centre observational study.^13^ We have utilized data from KIMS (Pfizer International Metabolic Database) to further elucidate this phenomenon.

## Patients and methods

### KIMS Database

KIMS is the Pfizer International Metabolic Database and pharmacoepidemiological survey of adult hypopituitary (GHD) patients treated with GH replacement therapy (Genotropin®). Enrolment into KIMS currently stands at approximately 12 000 patients from 28 countries. Following enrolment, patients are seen in local clinics at a frequency determined by the treating physician but with one visit per year being mandatory. At each visit, data are collected on specific case record forms and entered into a central database. The quality of data collection is monitored by clinical research representatives according to Good Clinical Practice Guidelines and the accuracy of data entry into the database is monitored by internal audit.

### Patients and study design

Lipoprotein profiles were measured prior to and after 12 months GH replacement therapy in 61 patients (30 males, 31 females) on stable maintenance statin therapy (statin group, SG) and in 1247 patients of similar mean age and range (608 males, 639 females) not on hypolipidaemic therapy (nonstatin group, NSG). All patients were enrolled between 1994 and 2004. Details of aetiological diagnosis, additional hormone replacement therapy, prevalence of diabetes mellitus and duration of statin use are provided in Table 1. The range of specific statins used and dose schedules are given in Table 2. It is noteworthy that diabetes mellitus was more prevalent in the SG group (18% *vs*. 4·4%) at baseline. Patients were either completely naïve to GH replacement or had not received GH for at least 6 months prior to the study and the study was restricted to patients with adult onset disease. Patients who developed diabetes mellitus during the first year of GH therapy or in the subsequent year were excluded from this analysis (3 on statins and 31 not on hypolipidaemic therapy). Doses of thyroid hormone replacement were constant throughout the period of observation.

**Table 1:** Aetiology of hypopituitarism, additional hormone replacement, prevalence of diabetes mellitus in statin group (SG) and nonstatin group (NSG) and duration of statin use in SG

Aetiology	SG (%)	NSG (%)
Pituitary adenoma	66·6	72·1
Craniopharyngioma	6·4	6·6
Other pituitary/hypopituitary tumours	5·1	1·6
Cranial tumours	3·0	0
Extracranial malignancy	0·6	0
Other causes acquired GHD	13·6	18·0
Idiopathic GHD	4·7	1·6
**Hormone replacement**		
TSH	70·8	68·8
ACTH	67·2	63·9
LH/FSH	78·3	78·7
ADH	23·4	16·4
**Diabetes mellitus**	4·4	18·0*
**Duration of statin therapy before entry into KIMS**†	2·6 ± 2·7 SD	

† Only patients who were treated with statins for at least 1 year before entry into KIMS were included.

* *P* < 0·0001. GHD, growth hormone deficiency.

**Table 2:** Prevalence and dose range of individual statin preparations in statin group (SG)

Statin preparation	Dose (mean ± SD; mg)	Dose prevalence (%)
Simvastatin	15·8 ± 8·1	52·0
Atorvastatin	14·4 ± 7·8	30·0
Pravastatin	31·6 ± 13·9	9·8
Lovastatin	17·5 ± 5	6·6
Fluvastatin	40	1·6

All patients had severe GHD as demonstrated by a peak serum GH response of < 9 mU/l (< 3 µg/l) during insulin, glucagon or arginine testing.

### Serum IGF-I assay

Until November 2002, serum IGF-I concentrations were determined by radioimmunoassay (RIA) after acid-ethanol precipitation of IGF binding proteins (Nichols Institute Diagnostics, San Juan Capistrano, CA). Thereafter, a chemiluminescence immunoassay (Nichols Advantage® System, Nichols Institute Diagnostics, San Juan Capistrano, CA) was introduced.^18^ Long-term reproducibility, measured during a time period of more than 1 year, showed a coefficient of variation (CV) of less than 9% in the concentration range of 130–850 µg/l (17·0–111 nmol/l) for both methods. The assay detection limit was 30 ng/ml (3·9 nmol/l). All measurements were performed in a single laboratory.

### Lipoprotein profiles

Serum concentrations of total cholesterol,^19^ HDL-cholesterol^20^ and triglycerides^21^ were measured as previously described^19^–^21^ and expressed in mmol/l. From these results serum concentrations of LDL-cholesterol were estimated using Friedewald's formula.^22^ All measurements were performed in a single laboratory.

Long-term reproducibility, measured during a time period of more than 1 year showed CV of < 3% in the concentration range of 4–6 mmol/l for total cholesterol, < 5% in range of 1–2 mmol/l for HDL-cholestrol, < 4% in range of 1–2 mmol/l for triglycerides and < 6% for LDL-cholestrol for range estimated from the above CV values.

### Statistical analysis

Baseline data and changes during GH replacement are expressed as means ± SD. Comparisons of non-normally distributed data were performed using the Wilcoxon rank sum test for paired and unpaired data and normally distributed data were compared using paired and unpaired Student's *t*-tests as appropriate. Statistical significance was accepted at *P* < 0·05. Correlations between baseline serum lipid measurements and changes after 12 months of GH replacement were determined using Pearson correlation coefficients.

## Results

### GH dose and serum IGF-I

Mean GH dose requirement was slightly but significantly lower in the SG group compared with the NSG group (SG *vs*. NSG, 0·32 ± 0·178 mg/day *vs*. 0·38 ± 0·197 mg/day; *P* = 0·0067).

The IGF-I SDS increased significantly from –1·4 ± 1·37 at baseline to 0·7 ± 1·39 (*P* < 0·001) after 12 months of GH treatment in the SG group and from –1·8 ± 1·75 to 0·4 ± 1·52 (*P* < 0·001) in the NSG group.

### Baseline serum lipoprotein profiles

Concentrations of serum total, LDL- and HDL-cholesterol and triglycerides prior to commencement of GH replacement therapy are provided in Table 3. Serum total and LDL-cholesterol were significantly higher in the NSG group (5·8 ± 1·15 *vs*. 5·2 ± 1·39 and 3·7 ± 1·03 *vs*. 3·1 ± 1·27 mmol/l, respectively; *P* < 0·0001) and serum triglycerides higher in the SG group (2·0 ± 0·73 *vs*. 1·8 ± 0·87 mmol/l; *P* < 0·02). Serum HDL-cholesterol concentrations were similar in the two groups.

**Table 3:** Measurements of serum total, low density lipoprotein (LDL)- and high density lipoprotein (HDL)-cholesterol and triglycerides at baseline and during 12 months GH replacement in statin group (SG) and nonstatin group (NSG) in (a) males (b) females (shown as means ± SD)

(a)				

	SG		NSG	
		
	Baseline	1 year	Baseline	1 year
Total cholesterol (mmol/l)	5·1 ± 1·24	4·6 ± 0·93	5·7 ± 1·16	5·3 ± 1·08
LDL-cholesterol (mmol/l)	3·1 ± 1·15	2·5 ± 0·87	3·7 ± 1·04	3·4 ± 0·97
HDL-cholesterol (mmol/l)	1·1 ± 0·34	1·1 ± 0·39	1·2 ± 0·33	1·2 ± 0·33
Triglycerides (mmol/l)	2·1 ± 0·75	2·1 ± 1·02	1·8 ± 0·87	1·8 ± 0·87

(b)				

	SG		NSG	
		
	Baseline	1 year	Baseline	1 year
Total cholesterol (mmol/l)	5·3 ± 1·53	4·9 ± 1·08	5·9 ± 1·13	5·6 ± 1·20
LDL-cholesterol (mmol/l)	3·1 ± 1·39	2·6 ± 0·81	3·7 ± 1·03	3·4 ± 1·08
HDL-cholesterol (mmol/l)	1·3 ± 0·29	1·4 ± 0·33	1·4 ± 0·40	1·4 ± 0·40
Triglycerides (mmol/l)	1·9 ± 0·71	1·8 ± 0·90	1·8 ± 0·86	1·8 ± 0·82

### Changes in serum lipoproteins during GH replacement

**Fig. 1:**
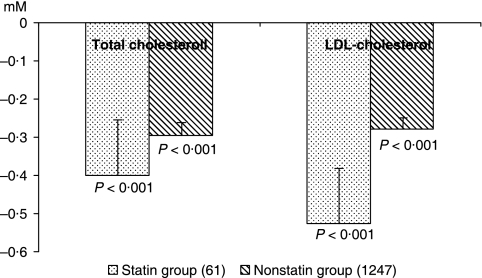
Decrement in serum total and LDL-cholesterol in statin group (SG) and nonstatin group (NSG) during 12 months GH replacement. Data are shown as mean ± SEM. *P < 0·0004, **P < 0·0001 *vs*. baseline.

**Fig. 2:**
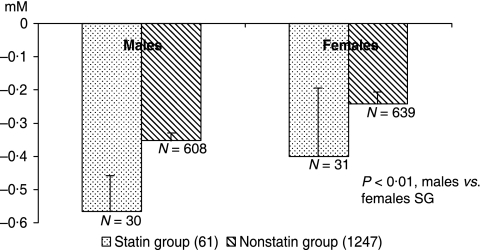
Decrement in serum total cholesterol in male and female patients in statin group (SG) and nonstatin group (NSG) during 12 months GH replacement. Data are shown as mean ± SEM. *P* < 0·01, males *vs*. females in SG.

**Fig. 3:**
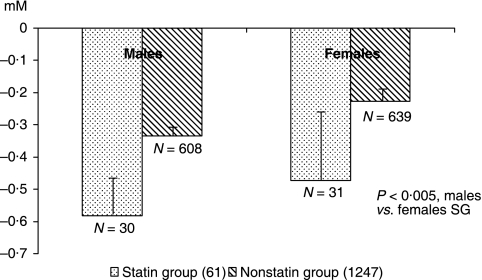
Decrement in serum LDL-cholesterol in male and female patients in statin group (SG) and nonstatin group (NSG) during 12 months GH replacement. Data are shown as mean ± SEM. *P* < 0·005, males *vs*. females in SG.

**Fig. 4:**
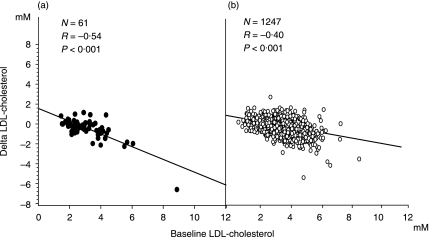
Relationship between baseline LDL-cholesterol and change in LDL-cholesterol in (a) statin group (SG), *R* = –0·54, *P* < 0·001 and (b) nonstatin group (NSG), *R* = –0·4, *P* < 0·001.

Serum total and LDL-cholesterol decreased significantly in both the SG and NSG groups during GH replacement (Table 3; Fig. 1). The decrement in both total and LDL-cholesterol was apparently greater in the SG group but this did not reach statistical significance. No significant changes in HDL-cholesterol or triglycerides were evident in either group (Table 3). A qualitatively similar pattern of change in total and LDL-cholesterol was seen in male and female patients in both groups but the decrements were significantly greater in male patients in the SG group (Table 3; Figs 2 and 3). A significant correlation between baseline measurements and decrements in serum total cholesterol during GH replacement was evident in both the SG and NSG groups (*R* = –0·51, *P* < 0·001; and *R* = –0·37, *P* < 0·001, respectively). A similar pattern was evident for LDL-cholesterol and this is demonstrated graphically in Fig. 4.

## Discussion

The observed decrements in serum total and LDL-cholesterol in this study are consistent with previous observations on the beneficial effects of GH replacement therapy derived from single-centre studies.^12^,^13^,^23^ In addition, we have demonstrated that patients already established on maintenance hypolipidaemic (statin) therapy derive additional benefit from GH therapy, the data concurring with previous preliminary observations.^13^ Previous analyses of the KIMS database have demonstrated that the decrement in serum cholesterol during GH replacement is proportional to baseline cholesterol measurements,^14^,^24^ a phenomenon which is not explained by simple regression to the mean.^14^ In this context, it is noteworthy that the SG patients in the present study had lower baseline total and LDL-cholesterol than the NSG patients but nonetheless demonstrated a statistically equivalent decrement during GH replacement. Indeed the changes in the SG patients were if anything more striking although the apparent difference failed to reach statistical significance. These findings raise the possibility that the effects of statins and GH replacement on serum cholesterol and LDL-cholesterol may be synergistic rather than simply additive. A particular strength of our study was the centralized measurement of lipoprotein profiles. However, it must be borne in mind that by virtue of the observational nature of the study we cannot be certain that statin therapy had been optimized in all patients or that compliance with therapy was absolute. Therefore we cannot exclude the possibility that intensification of statin therapy might have limited the extent to which GH replacement provided an additional hypolipidaemic effect.

The development of glucose intolerance may exert a confounding effect on the interpretation of longitudinal lipoprotein profile data. For this reason we excluded from our study all patients who developed diabetes mellitus during the period of study and extended this to a further 12 months after completion in order to eliminate the possibility that a diagnosis of diabetes mellitus had been missed in the period of observation. Similarly, maintenance of a constant thyroxine replacement dose was a requirement for inclusion in the analysis.

Statin therapy and GH replacement are likely to exert beneficial effects on serum total and LDL-cholesterol by increasing numbers of LDL-receptors on hepatocytes although the mechanism underlying this phenomenon differs between the two agents.^25^ Therefore an additive effect on cholesterol lowering would be predicted and has been confirmed by the present observations. It remains possible that a synergistic effect may operate and although we observed a numerically greater effect with combined statin and GH therapy, this did not reach statistical significance. Our data indicate that the effects of GH on serum cholesterol are complementary and at least additive to those of statin therapy and argue for an important and specific effect of GH replacement on cardiovascular risk in adult hypopituitary patients.
